# Activation of PPAR*γ* by Rosiglitazone Does Not Negatively Impact Male Sex Steroid Hormones in Diabetic Rats

**DOI:** 10.1155/2009/101857

**Published:** 2009-06-11

**Authors:** Mahmoud Mansour, Elaine Coleman, John Dennis, Benson Akingbemi, Dean Schwartz, Tim Braden, Robert Judd, Eric Plaisance, Laura Ken Stewart, Edward Morrison

**Affiliations:** Department of Anatomy, Physiology and Pharmacology, Auburn University, AL 36849, USA

## Abstract

Peroxisome proliferator-activated receptor gamma (PPAR*γ*) activation decreased serum testosterone (T) in women with hyperthecosis and/or polycystic ovary syndrome and reduced the conversion of androgens to estradiol (E2) in female rats. This implies modulation of female sex steroid hormones by PPAR*γ*. It is not clear if PPAR*γ* modulates sex steroid hormones in diabetic males. Because PPAR*γ* activation by thiazolidinedione increased insulin sensitivity in type 2 diabetes, understanding the long term impact of PPAR*γ* activation on steroid sex hormones in males is critical. Our objective was to determine the effect of PPAR*γ* activation on serum and intratesticular T, luteinizing hormone (LH), follicle stimulating hormone (FSH) and E2 concentrations in male Zucker diabetic fatty (ZDF) rats treated with the PPAR*γ* agonist rosiglitazone (a thiazolidinedione). Treatment for eight weeks increased PPAR*γ* mRNA and protein in the testis and elevated serum adiponectin, an adipokine marker for PPAR*γ* activation. PPAR*γ* activation did not alter serum or intratesticular T concentrations. In contrast, serum T level but not intratesticular T was reduced by diabetes. Neither diabetes nor PPAR*γ* activation altered serum E2 or gonadotropins FSH and LH concentrations. The results suggest that activation of PPAR*γ* by rosiglitazone has no negative impact on sex hormones in male ZDF rats.

## 1. Introduction

Peroxisome proliferator-activated receptors (PPARs) are a group of nuclear transcription factors which belong to the steroid receptor superfamily but are not activated by steroid hormones. Three PPAR isotypes have been identified and include PPAR*α* (NR1C1), PPAR*β* (NR1C2, *δ*, NUC-1, fatty acid-activated receptor (FAAR)), and PPAR*γ* (NR1C3). A large number of both endogenous (natural) and exogenous (synthetic) ligands activate either a single PPAR isoform or all isoforms, albeit with different binding affinities and specificities [1]. Among the important PPAR*γ* synthetic activators are the thiazolidinediones (TZDs) drugs often used in the treatment of type 2 diabetes. These include Avandia (rosiglitazone), Actos (pioglitazone), a combination drug, Avandamet (rosiglitazone and metformin), and Rezulin (troglitazone). Troglitazone was withdrawn from the market because of idiosyncratic liver toxicity. Activation of PPAR*γ* by TZDs increases insulin sensitivity and thus improves body glycemic control [2, 3].

PPARs are involved in a broad range of functions that include lipid homeostasis [2], tissue remodeling, angiogenesis, prostaglandin production [3], and steroidogenesis [4]. Additionally, PPARs also regulate inflammatory pathway by transrepression of transcription activity of proinflammatory transcription factors such as nuclear factor *κ*B (NF-*κ*B) [5]. Likewise, several data implicate PPARs in regulation of profibrotic [6–8] and oxidative stress responses in several cell types [9–11].

Support for the hypothesis that activation of PPARs, specifically PPAR*γ*, has an impact on sex steroid hormones action and/or production comes from several TZDs studies including two studies in male subjects [4, 12–22]. A study in healthy nondiabetic men showed that rosiglitazone treatment (8 mg/d for seven days) reduced the production rate of testosterone (T) and dihydrotestosterone (DHT) [14]. Similarly, rosiglitazone treatment of obese nondiabetic Zucker rats (0.01% wt/wt food admixture equivalent to 4 mg/kg/d for 36 days) reduced DHT but did not alter serum T [22].

Multiple studies using ovarian and other cell culture models support a steroidogenic role for PPAR*γ*. First, activation of PPAR*γ* with troglitazone, a TZD insulin sensitizer and putative PPAR*γ* agonist, inhibited aromatase cytochrome P450 activity, the enzyme critical in the conversion of androgens to estradiol (E2), in human adipose tissue [15] and in ovarian granulosa cells [20]. Similarly, activation of PPAR*γ* by troglitazone in vitro cultures of human and porcine granulosa cells inhibited progesterone production [4]. Troglitazone was also reported to competitively inhibit 3*β*-hydroxysteriod dehydrogenase (3*β*-HSD), the enzyme that catalyzes the conversion of pregnenolone to progesterone in the ovary [16]. Likewise, troglitazone was shown to inhibit androgen biosynthesis stimulated by combined LH and insulin in primary porcine thecal cell culture in a dose-dependent fashion [17].

In human adrenal NCI-H295R cells, an established in vitro model of steroidogenesis of the human adrenal cortex, both rosiglitazone and pioglitazone inhibited the activities of P450c17 and 3*β*-HSD type II both of which are key microsomal enzymes in the biosynthesis of all steroid hormones [18]. In diabetic women with polycystic ovarian syndromes (PCOs), a condition characterized by anovulatory androgen secretion, relatively high E2, and excessive LH production [23–25], treatment with the PPAR*γ* agonists rosiglitazone or pioglitazone improved insulin resistance and decreased hyperandrogenism in multiple studies (reviewed in [26–28]).

Toxicological studies showed that phthalate esters, used as plasticizers and stabilizers in several consumer products, activate PPAR*γ* [29], decrease key testicular steroidogenic enzymes [30] and reduce serum T production [31–34].

Although it is axiomatic that steroidogenic inhibition is a general characteristic of TZD compounds, involvement of PPAR*γ* and rosiglitazone in steroidogenic modulation under diabetic conditions remains unclear for several reasons. First, a study showed that the in vitro IC_50_ for rosiglitazone steroidogenic inhibition is far beyond its recommended therapeutic dose [13]. Second, a number of studies showed that TZDs, including rosiglitazone, directly inhibit 3*β*-HSDII and P450c17 steroidogenic enzymes independent of PPAR*γ* [13, 18]. Third, in the aforementioned male studies PPAR*γ* activation was not determined in parallel with sex hormone measurements. More importantly none of the research subjects used was diabetic where the steroidogenic effect of diabetes is an important component for evaluation of TZDs-PPAR*γ* activators. Finally, the treatment period used in the aforementioned male studies was short and varies between 7 and 36 days. Because of the above limitations, the objective of this study was to determine the link between relatively short term (8 weeks) activation of PPAR*γ* and the profile of T and E2 in male Zucker diabetic fatty rats (ZDFs) treated with a therapeutic dose of rosiglitazone. 

## 2. Materials and Methods

### 2.1. Animals and Treatments

Male ZDF (fa/fa) rats and their age-matched lean controls (ZDF lean, fa/+ or +/+ ) were obtained from Charles River Laboratories (Indianapolis, Ind, USA) at 6 weeks of age. The (fa/fa) ZDF rats lack a functional leptin receptor and become hyperphagic and diabetic when fed a high fat diet. Rats were maintained under standard housing conditions (constant temperature of 22°C, *ad libitum* food and water, and 12:12 hours light/dark cycles) at an AAALAC-accredited lab animal facility at the College of Veterinary Medicine, Auburn University. Rats were housed in pairs and assigned to three groups with 8 rats per group. Lean nondiabetic group (group 1); ZDF rats randomly assigned to ZDF untreated group (group 2) and ZDF group treated with rosiglitazone (group 3). Lean rats were fed regular rat chow whereas ZDF rats in group 2 and 3 were fed Purina 5008 modified rat chow (Purina Mills, Richmond, Ind, USA). Rosiglitazone maleate (generously provided by GlaxoSmithKline, USA) was dissolved in 0.5% carboxymethylcelluose and administered daily via oral gavage at 3 mg/kg/d/rat starting at week 7 of age for 8 weeks. Rats in groups 1 and 2 received 0.5% carboxymethylcelluose vehicle. All rats were weighed and blood glucose was monitored from the tail vein weekly using an ACCU-CHEK glucose meter (Roche Diagnostics Co. Indianapolis, Ind, USA). Diabetes was confirmed by two consecutive measurements of blood glucose of > 200 mg/dl. All animal procedures were approved by the Institutional Animal Care and Use Committee at Auburn University.

### 2.2. Necropsy and Tissue Collection

Rats were sacrificed by deep anesthesia with pentobarbital (50 mg/kg intraperitoneal, IP) followed with decapitation. Testes were excised, and sampled for histopathology, RNA extraction, and intratesticular T assay. Visceral epididymal fat and prostate were collected for use as positive sources for PPAR*γ* expression in real-time PCR analysis. Tissues intended for RNA and hormone analysis were immediately frozen in liquid nitrogen and transferred to −80°C until processing. Trunk blood was collected for serum isolation and stored at −30°C prior to hormone analysis.

### 2.3. Total RNA Isolation

Total RNA was isolated using TRIzol reagent (Invitrogen-Life Technologies Inc., Carlsbad, Calif, USA), according to the manufacturer's instructions and as described previously in our laboratory [35]. Briefly, RNA concentrations were determined at 260 nm wavelength and the ratio of 260/280 was obtained using UV spectrophotometry (DU640, Beckman Coulter Fullerton, Calif, USA). RNA samples were treated with DNase (Ambion Inc.) to remove possible genomic DNA contamination and samples with 260/280 ratio of ≥1.8 were used.

### 2.4. Real-Time PCR and Agarose Gel Electrophoresis

Real-time PCR was used to determine expression of testicular PPAR*γ* mRNA and to quantify changes in mRNA level. Quantitative real-time PCR analysis was performed in 25 *μ*L reaction mixture containing RT^2^ Real-Time SYBR/Fluorescein Green PCR master mix with final concentrations of 10 mM Tris-Cl, 50 mM KCL, 2.0 mM MgCl_2_, 0.2 mM dNTPs, and 2.5 units of HotStart Taq DNA polymerase (Super Array Bioscience Corporation, Frederic, Md, USA). The reaction was completed with addition of 1 *μ*L first strand cDNA transcribed from 2 *μ*g total RNA, and 0.2 mM RT^2^ validated PCR primers for PPAR*γ* or GAPDH house keeping gene (Super Array Bioscience). Samples were run in 96-well PCR plates (Bio-Rad, Hercules, Calif, USA) in duplicates, and the results were normalized to GAPDH expression. The amplification protocol was set at 95°C for 15 minutes, and 40 cycles each at (95°C for 30 seconds, 55°C for 30 seconds, and 72°C for 30 seconds) followed by a melting curve determination between 55°C and 95°C to ensure detection of a single PCR product. Real-time PCR products at the end of each assay were combined for each treatment group and stored at −30°C for viewing on agarose gel electrophoresis. Verification of PCR product was confirmed by determination of expected band size and sequence analysis as we described previously [35]. The resulting sequences were matched with previously published rat sequences in Genbank (accession number NM_013124 for PPAR*γ*) using Chromas 2.31 software (Technelysium Pty ltd, Tewantin Qld 4565, Australia). RNA templates from white adipose tissue and prostate were used to generate standard curves for PPAR*γ* and GAPDH using 10-fold dilutions. Curves were made by plotting threshold cycle (*C*_*t*_ value) for each dilution versus the log of the dilution factor used. Relative differences in expression (fold increase or decrease) were calculated as described previously [36]. Pearson correlation coefficients (*r* values) for standard curves were between 0.98 and 0.99, and amplification efficiency was considered 100%.

### 2.5. Immunohistochemistry (IHC)

Immunolocalization of PPAR*γ* by IHC was performed as described previously by our laboratory [35]. Briefly, cross sections from testes were fixed in 4% paraformaldehyde for 48 hours, embedded in paraffin, and cut at 5 *μ*m thickness. Sections were also fixed in Bouin's fixative (BioSciences) for staining with hematoxylin and eosin. Mounted sections were deparaffinized in Hemo-D (Scientific Safety Products) and hydrated in distilled water. Antigen retrieval was performed by heating in citrate buffer. Sections were incubated in 5% normal goat serum containing 2.5% BSA to reduce nonspecific staining. PPAR*γ* was detected with mouse anti-PPAR*γ* monoclonal antibody (Santa Cruz: sc7273; diluted 1:80 in blocker) and the antibody-antigen complexes were visualized with Alexa 488-conjugated goat antimouse IgG (Molecular Probes). Sections were examined with a Nikon TE2000E microscope and digital images were made with an attached Retiga EX CCD digital camera (Q Imaging, Burnaby, BC, Canada).

### 2.6. Hormonal Assays

Total serum T (intraassay coefficient of variation (CV) was 4.3%), E2 (intraassay CV was 2.3%), and intratesticular (intraassay CV was 6%) level were determined by radioimmunoassay (RIA) using kits from Siemens Medical Solutions Diagnostics (Los Angles, Calif, USA) according to manufacturer's instructions. For intratesticular T, 100 mg of testicular tissue was homogenized in 500 *μ*L Tris-PBS buffer (0.01 M Tris-HCl; pH 7.4) in plastic tubes. An additional 500 *μ*L was added and the homogenate was mixed with eight volumes of diethyl ether. The mixture was then vigorously vortexed and the aqueous phase quickly frozen in a dry ice bath (70% ethanol; dry ice). Extracts were subsequently air dried (warm bath at approximately 50°C under the hood) and samples were subsequently resuspended in 500 *μ*L PBS-buffer. 50 *μ*L of 1:10 diluted sample were used in the COAT-A-COUNT radioimmunoassay and counted in a Cobra D5005-gamma counter (Packard Instrument Co., Downers Grove, Il, USA). All samples were quantified in duplicates in a single assay. FSH and LH were determined by radioimmunoassay at the Endocrine Laboratory, Fort Collins, Colorado State University.

### 2.7. Serum Adiponectin

Total serum adiponectin concentration was assayed using a sandwich ELISA method (Millipore Corporation, Billerica, Mass, USA) per manufacturer's instructions. The intraassay CV was 1.1% to 1.3%.

### 2.8. Statistical Analysis

Analysis of real-time PCR data was performed using a modification of the delta delta *C*_*t*_ method (ΔΔ*C*_*t*_). Δ*C*_*t*_ calculated from real-time PCR data were subjected to analyses of variance using Sigma Stat statistical software (Jandel Scientific, Chicago, IL). Hormonal data were subjected to analysis of variance. Treatment groups with means significantly different (*P* < .05) from controls were identified using Dunnett's test. When data were not distributed normally, or heterogeneity of variance was identified, analyses were performed on transformed data or ranked data. 

## 3. Results

### 3.1. Blood Glucose and Body Weight

The ZDF rats fed Purina 5008 high fat diet in groups 2 and 3 became diabetic by week 7 of age. By week 15 of age the mean blood glucose concentration, determined shortly before necropsy, was >600 mg/dl (above glucose meter range) in ZDF-untreated controls (group 2) versus 123 ± 1.7 mg/dl in lean nondiabetic controls (group 1) and 163.6 ± 17.7 in ZDF rats treated with rosiglitazone (group 3). Rats in all three experimental groups gained weight over time irrespective of treatment. The ZDF-untreated rats mean body weight at week 15 was not significantly different from lean nondiabetic (382.75 ± 10.94 versus 365 ± 8.2 gm, resp.; *P* > .05). In contrast, the mean body weight of ZDF treated rats (group 3) was more than 40% above that of ZDF untreated rats at week 15 (638.6 ± 14.67 versus 382.75 ± 10.9 gm, *P* < .001).

### 3.2. Serum Adiponectin

Serum adiponectin was determined to confirm PPAR*γ* activation [37]. As expected, treatment with rosiglitazone increased serum adiponectin significantly in ZDF-treated compared with ZDF-untreated rats (46.33 ± 2.83 versus 12.71 ± 0.69 *μ*g/mL, *P* < .001) or lean nondiabetic untreated rats (46.33 ± 2.83 versus 17.13 ± 0.95 *μ*g/mL, *P* < .001). In contrast, adiponectin was significantly reduced by diabetes in ZDF-untreated compared with lean nondiabetic rats (17.13 ± 0.95 versus 12.71 ± 0.69 *μ*g/mL, *P* < .05).

### 3.3. Real-Time PCR, Agarose Gel Electrophoresis and IHC

**Figure 1:**
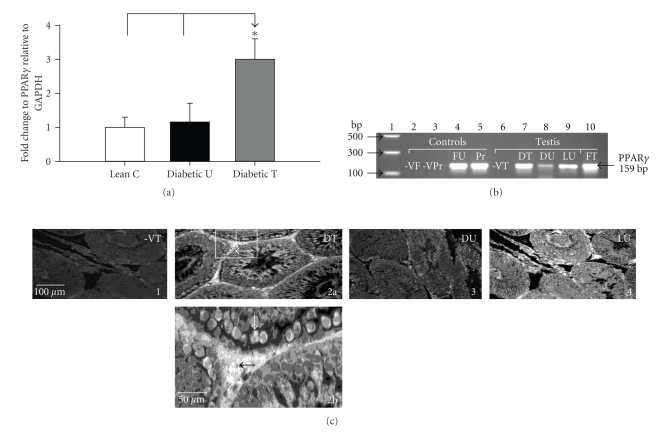
(a) Real-time PCR analysis of testicular PPAR*γ* mRNA levels in lean nondiabetic controls (*Lean C*), Zucker diabetic fatty (ZDF) untreated (*Diabetic U*), and ZDF rats treated with rosiglitazone (*Diabetic T*). Data are expressed as mean ± SE. *n* = 8 per group, *P < .05. (b) Agarose gel (2%) showing real-time PCR products generated in (a). Lane 1, DNA markers, lanes 2-3, RNA templates (instead of cDNA) from fat (-VF) and prostate (-VPr) as negative controls. Lanes 4-5, fat from untreated rats (FU) and prostate (Pr) as positive controls. Lanes 6–9, testicular PCR products from RNA negative controls (-VT), *diabetic treated (DT)*, *Diabetic untreated (DU)*, and *lean untreated (LU)* rats. Lane 10, fat from ZDF-treated rats (FT). (c) Representative IHC of PPAR*γ* protein in the testis of DT (panel 2a with insert box magnified in 2b), DU (panel 3), and LU rats (panel 4). Panel 1, -VT = negative control testis section (minus primary antibody. Arrows indicate PPAR*γ* localization in spermatogonia and in Leydig cells.

Real-time PCR data showed that PPAR*γ* mRNA was expressed in the testis and was upregulated by more than two folds with rosiglitazone treatment (Figures 1(a) and 1(b)). As shown in the IHC data (Figure 1(c)) PPAR*γ* protein was specifically localized in Leydig cells located in the interstitial space between the seminiferous tubules and in spermatocytes within the inside of seminiferous tubules basement membranes. 

### 3.4. Testicular Morphology and Histopathology

**Figure 2:**
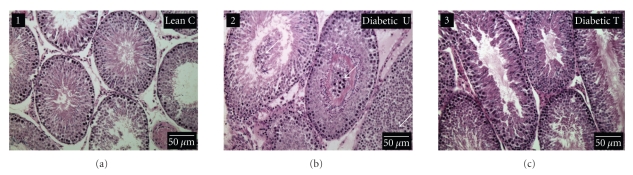
Representative photomicrographs of hematoxylin-eosin-stained sections of testis of nondiabetic Zucker lean control (*Lean C*, panel 1), Zucker diabetic fatty (ZDF) untreated (*Diabetic U*, panel 2), and ZDF rats treated with rosiglitazone (*Diabetic T,* panel 3). Arrows indicate germ cells strewn in the lumen of seminiferous tubules.

Detachment and disorganization of germ cells was evident in ZDF-untreated rats but there was no significant changes in the overall morphology of seminiferous tubules (Figure 2). Likewise, treatment did not alter seminiferous tubules morphology but desirably reversed germ cells sloughing (ZDF-treated in panel 2 versus ZDF-untreated in panel 3). 

### 3.5. Serum and Intratesticular T

**Figure 3:**
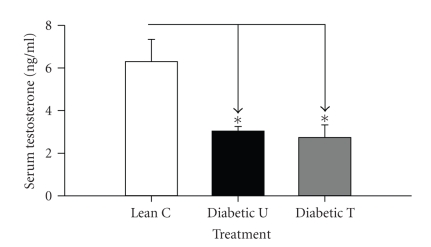
Serum T level in Zucker lean nondiabetic controls (*Lean C*), Zucker diabetic fatty- (ZDF-) untreated (*Diabetic U*), and ZDF rats treated with rosiglitazone (*Diabetic T*). Data are expressed as means ± SE. *n* = 8 per group. *P < .05.

**Figure 4:**
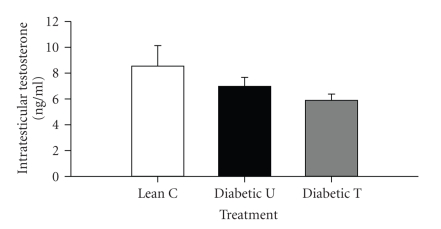
Intratesticular T level in Zucker lean nondiabetic controls (*Lean C*), Zucker diabetic fatty- (ZDF-) untreated (*Diabetic U*), and ZDF rats treated with rosiglitazone (*Diabetic T*). *n* = 8 per group. Data are expressed as means ± SE.

Total serum and intratesticular T were not significantly altered by PPAR*γ* activation in the testis (ZDF-treated versus ZDF-untreated, *P* > .05) (Figures 3 and 4). As expected total serum T was significantly lowered by diabetes (ZDF-untreated or treated versus lean nondiabetic, *P* < .05) (Figure 3). Surprisingly, the significant reduction in total serum T in diabetic rats was not associated with a corresponding significant reduction in intratesticular T production (Figure 4). A trend toward lower intratesticular T in ZDF versus lean rats was apparent irrespective of rosiglitazone treatment.

### 3.6. Serum E2

**Figure 5:**
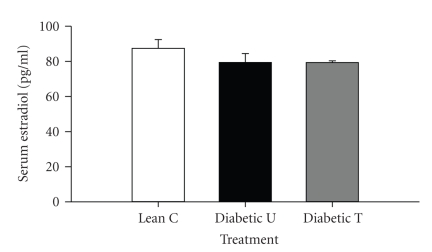
Serum E2 level in Zucker lean nondiabetic controls (*Lean C*), Zucker diabetic fatty- (ZDF-) untreated (*Diabetic U*), and ZDF rats treated with rosiglitazone (*Diabetic T*). *n* = 6 per group. Data are expressed as means ± SE.

Neither diabetes nor PPAR*γ* activation with rosiglitazone adversely altered serum E2 (Figure 5). 

### 3.7. Serum FSH and LH

**Figure 6:**
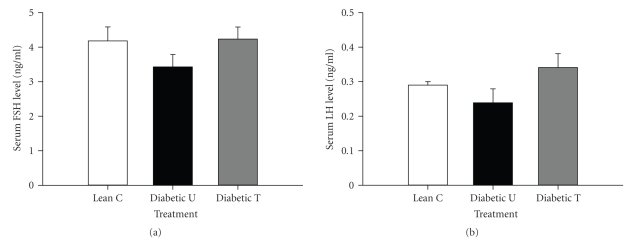
Serum FSH (a) and LH (b) in Zucker lean nondiabetic controls (*Lean C*), Zucker diabetic fatty- (ZDF-) untreated (*Diabetic U*), and ZDF rats treated with rosiglitazone (*Diabetic T*). *n* = 7-8 per group. Data are expressed as means ± SE.

Serum FSH and LH were not significantly altered by activation of PPAR*γ* with rosiglitazone and/or by diabetes (*P* > .05). The ZDF-untreated rats, however, showed a trend for lower FSH and LH (ZDF-untreated versus lean nondiabetic) and treatment with rosiglitazone reversed this tendency (ZDF-treated versus ZDF-untreated rats) (Figures 6(a) and 6(b)).

## 4. Discussion

Rosiglitazone and other TZDs were shown to decrease hyperandrogenemia in women with PCOs and repress major steriodogenic enzymes (reviewed in [26]). Although the in vitro steroidogenic repression potency of rosiglitazone was ranked intermediate between troglitazone and pioglitazone [13], its in vivo impact on sex steroids in diabetic subjects was unknown. Specifically, information on how changes in serum steroids in male diabetic subjects relate to changes in PPAR*γ* activity was lacking. This study documents the link between testicular PPAR*γ* activation with the anti-diabetic drug rosiglitazone and male sex hormone profile under diabetic conditions. Daily oral gavage of ZDF rats with rosiglitazone activated PPAR*γ* in the testis and normalized testicular germ cell derangement seen in diabetic testis. The stimulation of systemic and testicular PPAR*γ* activity by rosiglitazone, however, did not significantly alter the concentrations of gonadotropins LH and FSH, sex steroid T (both total serum and intratesticular) or serum E2.

Systemic activation of PPAR*γ* in this study was evident by the reversal of hyperglycemia, increased serum adipocytokine adiponectin, and by weight gain in ZDF-treated rats. These effects are considered biological signatures for rosiglitazone-induced PPAR*γ* activation in typically body fat depots [38, 39]. Specific activation of testicular PPAR*γ* shown here is consistent with other unrelated studies that showed the presence of this receptor in rat testis [40] and its modulation by synthetic chemicals such as phthalate esters [34, 41, 42]. Because PPAR*γ* can also be activated by endogenous ligands such as polyunsaturated fatty acids from diet sources and by metabolites of arachidonic acid [43], contribution of these ligands to the observed up regulation of testicular PPAR*γ* mRNA and protein in ZDF-treated rats could not be ruled out.

Compared to nondiabetic lean rats, ZDF-untreated rats showed consistent structural disorganization of germinal epithelium evident by abnormal accumulation of germ cells in the lumen of seminiferous tubules. This effect likely resulted from diabetes-induced oxidative stress previously recognized in diabetic rat testis [44]. Interestingly, the positive staining of germ cells for PPAR*γ* protein in IHC data suggests that rosiglitazone crosses the blood-testis barrier to enter the adluminal compartment of the seminiferous tubules resulting in activation of PPAR*γ* and reversal of germ cell sloughing. This novel effect is possibly mediated by the reported functional property of PPAR*γ* to ameliorate oxidative stress [11].

As previously known [45], diabetes in this study significantly lowered serum T. Rosiglitazone treatment, however, neither restored nor reduced serum T in ZDF-treated rats. Paradoxically, the diabetes-induced reduction in serum T in ZDF-treated and untreated rats were not associated with a corresponding significant drop in intratesticular T production, or androgen receptor expression (data not shown). Although unexpected, this finding was consistent with the nonsignificant changes observed in serum LH concentration. The unaltered intratesticular T production contrasted with significantly lowered serum T concentration may reflect a reduction in sex hormone binding globulin (SHBG) essential for T transportation in blood [46]. Reagents for determination of rat SHBG are not currently available and attempts to quantify rat SHBG using a human ELISA kit were not successful because of reagents incompatibility.

Rosiglitazone treatment reversed obesity-induced reduction of T intermediate steroid hormone precursor 17-hydroxyprogesterone in the genetically related obese but nondiabetic Zucker rats [22]. In the same study, however, rosiglitazone treatment did not alter total serum T. Compared with the above findings the data in our study allow for differentiation of diabetes steroidogenic effects (ZDF-untreated versus lean nondiabetic rats) from those of PPAR*γ* activation (ZDF-treated versus ZDF-untreated rats) but does not permit a way for separation of obesity effects from those of diabetes. The lack of significant change in total serum T in the above study was nevertheless consistent with our findings. In both the aforementioned and our study a tendency toward lowered serum T production was observed in ZDF and Zucker obese versus lean littermates irrespective of treatment. A normal transient T reduction was reported in 2-4 months old Zucker obese rats [47]. The age of rats at the point of serum collection in this study was 3.75 months and thus the lowered T observed in the ZDF versus lean rats could be a reflection of the above observation in these two genetically related rat models.

Studies that support a steroidogenic regulatory role for PPAR*γ* in women treated with TZDs and in vitro ovarian cell culture models have produced contrasting results as outlined in several reviews [1, 21, 26, 27]. While some in vitro studies showed that rosiglitazone and other TZDs such as troglitazone could inhibit steroidogenic enzymes independent of PPAR*γ* activation [4, 13, 16–18, 20], an inhibitory effect of rosiglitazone was not reflected in total T and E2 concentrations in ZDF-treated rats in our study. In summary, our data show that PPAR*γ* activation with rosiglitazone for eight weeks had no negative impact on total sex hormone concentrations in diabetic male rats. This finding is firmly in line with studies that showed strong TZD-PPAR*γ* activation [48, 49] but weaker in vitro steroidogenic inhibitory effects of rosiglitazone [13].
